# Pittsburgh as a High Risk Population: The Potential Savings of a Personalized Dental Care Plan

**DOI:** 10.1155/2016/3105417

**Published:** 2016-02-23

**Authors:** Andrew J. Ng, Alexandre R. Vieira

**Affiliations:** Department of Oral Biology, University of Pittsburgh School of Dental Medicine, 614 Salk Hall, 3501 Terrace Street, Pittsburgh, PA 15261, USA

## Abstract

*Objectives*. Little evidence exists for the current standard of two annual preventative care visits. The purpose of this study was investigate this claim by modeling the potential savings of implementing a personalized care plan for high risk individuals in the Pittsburgh region.* Methods*. Using radiographs from 39 patients in the University of Pittsburgh Dental Registry and DNA Repository database, two models were created to analyse the direct savings of implementing a more aggressive preventative treatment plan and to view the longitudinal cost of increased annual yearly visits.* Results*. There is a significant decrease (*p* < 0.001) between original and modeled treatment cost when treatment severity is reduced. In addition, there is a significant decrease in adult lifetime treatment cost (*p* < 0.001) for up to four annual visits.* Conclusions*. Patients in high risk populations may see significant cost benefits in treatment cost when a personalized care plan, or higher annual preventative care visits, is implemented.

## 1. Introduction

Currently, the standard of care is for patients to receive insurance coverage for two yearly preventative care visits to their dentist; however, there is very little evidence that this number of annual visits supports adequate oral health status. Of the limited number of research studies conducted on the subject, survey studies investigating the ideal time for dental checkups revealed that in adults 16 or older, 18 months is the ideal time between visits, whilst in populations where water is treated with fluoride, this period may be extended even longer [1]. Analysis of risk factors such as diabetes or smoker status may help dentists decide what this annual visit frequency should be; it has been shown that the presence of certain risk factors may increase the risk for tooth loss [2]. Furthermore, patients with more than one risk factor may need more than two annual preventative care visits to reduce tooth loss [3]. With such variation of oral health status in age, health markers, and environmental factors, it appears that personalized dental care improves the overall treatment experience of patients.

Pittsburgh and the surrounding Appalachia region is an ideal place to study long term dental care effects due to a generally lower socioeconomic status compared to the broader United States. It has been noted that this, in addition to the presence of rural communities, has contributed to disparities in oral health status [4]. In addition, individuals being treated at the University of Pittsburgh Medical Center have displayed some of the worst health indicators in the country. The University of Pittsburgh School of Dental Medicine keeps health records and biological samples of consenting patients seeking treatment at the dental school through the Dental Registry and DNA Repository (DRDR). This project serves as a database to support genomic and data analysis studies through the use of patient information. This study aimed to model the potential cost benefits for implementing a personalized dental care plan for patients in this high risk location by analysing radiographic information obtained from a small population of subjects in this DRDR database.

## 2. Methods

Full mouth series and panoramic radiographs for 39 patients in the DRDR database were obtained and analysed for the presence of fourteen of the most common dental procedures, including amalgam restorations, root canal therapy, and extraction cases. All patients at the University of Pittsburgh School of Dental Medicine are invited to be a part of the DRDR study (University of Pittsburgh Institutional Review Board Approval #0606091); those that give written consent have electronic health information analysed from their dental records. Prices for these procedures were obtained from the University of Pittsburgh School of Dental Medicine financial department and applied to treatment totals for these patients. Preliminary analysis of these data included male and female spending trends and a look at the distribution of patient spending on the analysed procedures. It was noted that almost half of all extraction procedures were wisdom tooth removals; since wisdom tooth anomalies are often more impacted by genetics rather than home care, subsequent data analysis removed wisdom tooth extractions from treatment totals.

Two models were created and applied to this sample population. The first model sought to determine the effect of reducing the severity of disease through increased preventative care visits whilst maintaining the same number of procedures performed. The purpose was to demonstrate the direct savings of implementing a more intensive preventive regimen presumably for individuals at higher risk for oral disease over the traditional two yearly visits. This was done by categorizing the analysed procedures by severity of invasiveness and trauma/pain to the patient and reducing the severity of each procedure accordingly whilst adjusting the prices. For instance, an extracted mandibular first permanent molar due to decay would have received at least one single surface restoration before being extracted. In cases where signs of endodontic treatment were present, the assumption was that during the lifetime of the lost tooth, individuals had a simpler restoration before having a more complex restoration after endodontic treatment. This was based on the tooth loss trends analysed in Vieira et al.'s paper [2]. The second model aimed to predict the lifetime cost of treatment for these high risk individuals and determine the financial savings of increased annual visits, thus ideally eliminating the presence of disease and the need for treatment. Because radiographs only show a one-time snapshot of patient treatment, it is difficult to quantify the amount of money spent on procedures over many years. In order to analyse the overall impact of increased annual preventative care visits, a longitudinal model was created to compare the lifetime cost of treatment and yearly preventative care visits with no additional procedural cost. This was done by anticipating the progression of treatment through different categories of severity, summing the cost of all the procedures required to reach that point. Since the average age of individuals was 45, annual treatment costs were calculated from years 18 to 45. The choice of four and six preventive dental visits was arbitrary (could have been three and five) and they were chosen based on the rationale of duplicating or triplicating the classic recommendation of biannual preventive dental visits.

Statistical analyses of these data included a paired 2 sample and 1 sample *t*-tests and 95% confidence intervals for graphical representations to determine significance of data. All statistical tests of significance were carried out at the *α* = 0.05 level.

## 3. Results

**Figure 1:**
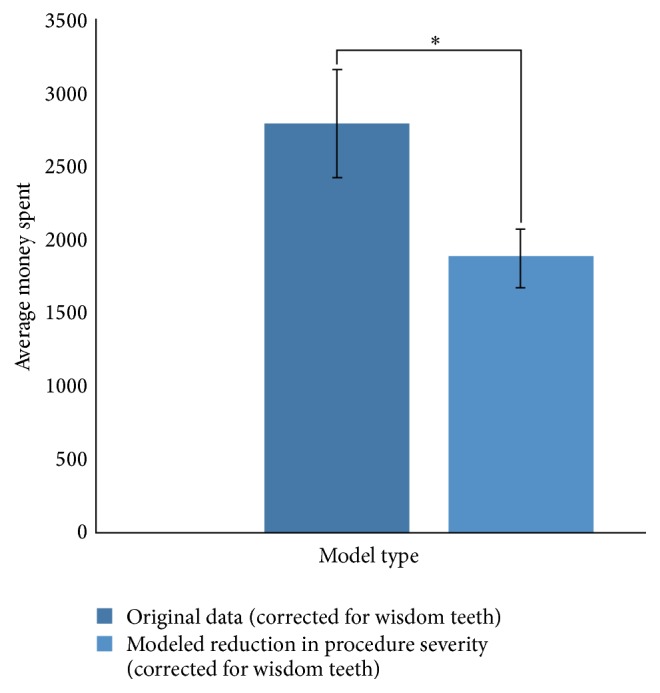
A comparison of average money spent on treatment between original and modeled data when disease severity is reduced but the number of procedures undertaken remains constant. The error bars are 95% confidence intervals. *∗* indicates significance at *α* = 0.05.

Statistical analysis using a paired *t*-test revealed that according to the first model, there was a significant difference (*p* < 0.001) between the amount of money spent on treatment in the original sample population and the modeled population (Figure 1). It appears that there is a significant decrease in the amount of money spent on treatment in the modeled population compared to the original sample population.

**Figure 2:**
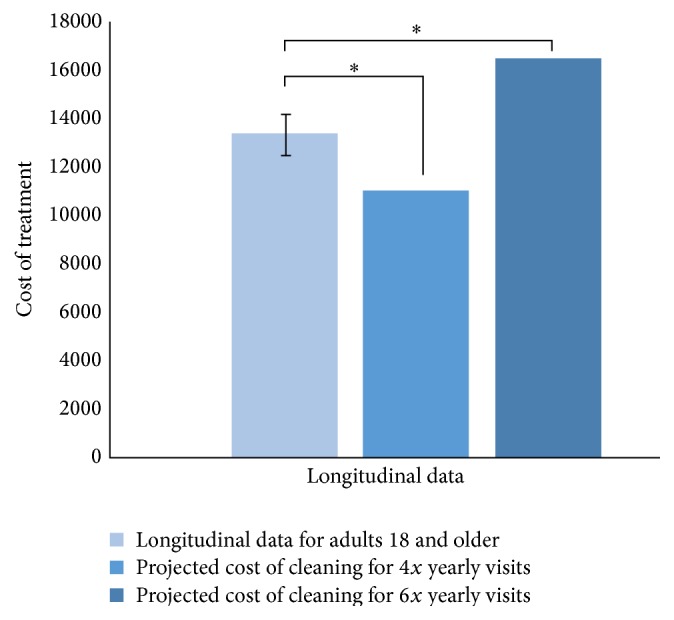
A model of longitudinal projection of treatment cost from 18–45-year-olds using patient data from the DRDR database, compared with the cost of 4 and 6 yearly preventative care visits over the same period (corrected for wisdom tooth removal). *∗* indicates significance at *α* = 0.05.

Analysis of the second model using a one sample *t*-test showed a significant (*p* < 0.01) difference between the projected cost of maintaining oral health treatment with four annual preventative care visits and the longitudinal price model for patients (Figure 2). In addition, a similar *t*-test analysis showed a significant (*p* < 0.01) difference between the projected cost of six annual dental visits and the longitudinal price model. This figure shows that there is a significant decrease in cost when comparing the sample population cost with 4 annual visits and a significant increase in cost when comparing the same population with 6 annual visits.

## 4. Discussion 

Pittsburgh and the surrounding Appalachia region contain patients with higher risk factors compared to those of other regions in the United States. Analysis shows that these high risk patients can significantly reduce the amount of money spent on dental procedures if disease severity is reduced through increased annual visits to the dentist. Under the model used in this analysis, up to 5 yearly visits would be supported before the cost for annual cleanings over 27 years would become significantly greater than the existing cost of treatment. Thus, it appears that the current two yearly preventative care visits are not cost-effective for high risk patients for the number of treatments needed to be performed. Under a personalized dental care system, individuals that are deemed to be of higher risk could benefit from increased annual preventative care visits. The implications for patient care and well-being are extensive. If these high risk individuals participated in increased yearly visits, perhaps patients could better manage and prevent oral diseases, thus leading to less pain and chair time involved in more extensive and invasive procedures as well as less money spent. The benefits of such a system would show increasing significance with older age; maintenance of natural tooth structures has been found to be the main predictor of masticatory performance in dentate adults [5].

This study was very limited in the scope of the analysis; the main underlying assumption of this study was that disease severity is correlated with treatment cost. This was not true for all procedures; it was discovered that extractions, which were considered to be a treatment option for patients with the most severe occurrence of disease, were amongst the cheapest procedures. In addition, analysis of this high risk in Pittsburgh population says nothing about patients who have little or no risk factors. Further studies could further investigate whether or not low risk individuals see similar cost benefits with decreased annual preventative care visits.
